# Supplementary material for: Ubiquitous quantum scarring does not prevent ergodicity
Source: Nat Commun. 2021 Feb 8;12:852. doi: 10.1038/s41467-021-21123-5 (PMC7870831; doi:10.1038/s41467-021-21123-5)
Supplement: Supplementary file 1 — Supplementary Information [file 41467_2021_21123_MOESM1_ESM.pdf]

Ubiquitous quantum scarring does not prevent ergodicity
Supplementary Information

Saúl Pilatowsky-Cameo,[1] David Villaseñor,[1] Miguel A. Bastarrachea-Magnani,[2,3]
Sergio Lerma-Hernández,[4] Lea F. Santos,[5] and Jorge G. Hirsch[1]
[1]*Instituto de Ciencias Nucleares, Universidad Nacional Autónoma de México, Apdo. Postal 70-543, C.P. 04510, CDMX, Mexico*
[2]*Department of Physics and Astronomy, Aarhus University, Ny Munkegade, DK-8000 Aarhus C, Denmark*
[3]*Departamento de Física, Universidad Autónoma Metropolitana-Iztapalapa, San Rafael Atlixco 186, C.P. 09340, CDMX, Mexico*
[4]*Facultad de Física, Universidad Veracruzana, Circuito Aguirre Beltrán s/n, Xalapa, Veracruz 91000, Mexico*
[5]*Department of Physics, Yeshiva University, New York, New York 10016, USA*

Supplementary Note 1:
Classical and quantum chaos in the Dicke model

The Dicke model displays regular and chaotic behavior [1–8]. For the parameters selected in the main text ($\omega = \omega_0 = 1, \gamma = 2\gamma_c$), the dynamics are regular up to $\epsilon \approx -1.6$ [7], then there is a mixed region of regularity and chaos up to $\epsilon \approx -0.8$, after which strong chaos sets in. The onset of chaos is illustrated in Supplementary Fig. 1 for the classical limit (a)-(b) and for the quantum domain (c)-(d).

Supplementary Fig. 1 (a) shows the percentage of chaos defined as the ratio of the number of chaotic initial conditions, determined by the Lyapunov exponent, over the total number of initial conditions for a very large sample. The percentage is presented as a function of the rescaled energy ϵ and the coupling strength γ. Following the vertical red dashed line marked at $\gamma = 2\gamma_c$, one sees that energies $\epsilon \sim -0.5$ are already deep in the chaotic region (light color). This is confirmed in Supplementary Fig. 1 (b), where the Poincaré section for $\epsilon = -0.5$ exhibits hard chaos, that is, all chaotic trajectories cover the entire energy shell densely and have the same positive Lyapunov exponent.

Supplementary Fig. 1 (c) displays the distribution $P(s)$ of the spacings s between nearest-neighboring unfolded energy levels. The eigenvalues of quantum systems whose classical counterparts are chaotic are correlated and repel each other. In this case, $P(s)$ follows the Wigner surmise [9], as indeed seen in Supplementary Fig. 1 (c).

In Supplementary Fig. 1 (d), we show the quantum survival probability, $S_P(t) = |\langle \mathcal{R}_\epsilon | e^{-i\hat{H}_D t} | \mathcal{R}_\epsilon \rangle|^2$ for a Gaussian ensemble of random initial states $|\mathcal{R}_\epsilon\rangle = \sum_k c_k \, |E_k\rangle$ whose components $|c_k|^2$ were generated through a random sampling (see Methods) and are centered at energy $\epsilon = -0.5$ in the chaotic region [10, 11]. The survival probability of individual random states are shown with gray solid lines, their ensemble average with an orange solid line, the running time average with a blue solid line, which overlaps with a green line that represents an analytical curve derived from the Gaussian orthogonal ensemble of the random matrix theory [10, 11]. The asymptotic value of $S_P(t)$ is shown with a horizontal red dashed line. The green and blue curves exhibit a dip below the saturation value of the quantum survival probability known as correlation hole, which is a dynamical manifestation of spectral correlations. It contains more information than the level spacing distribution $P(s)$, since in addition to short-range correlations, it captures also long-range correlations [10, 12–15]. We verified that most coherent states from the chaotic region develops the correlation hole. Exceptions to this pattern are the states very close to unstable periodic orbits of relatively short periods [11].

The four panels of Supplementary Fig. 1 leave no doubt that the Dicke model reaches a limit of very strong chaos. This is the region for which our analysis of the quantum scars is developed.

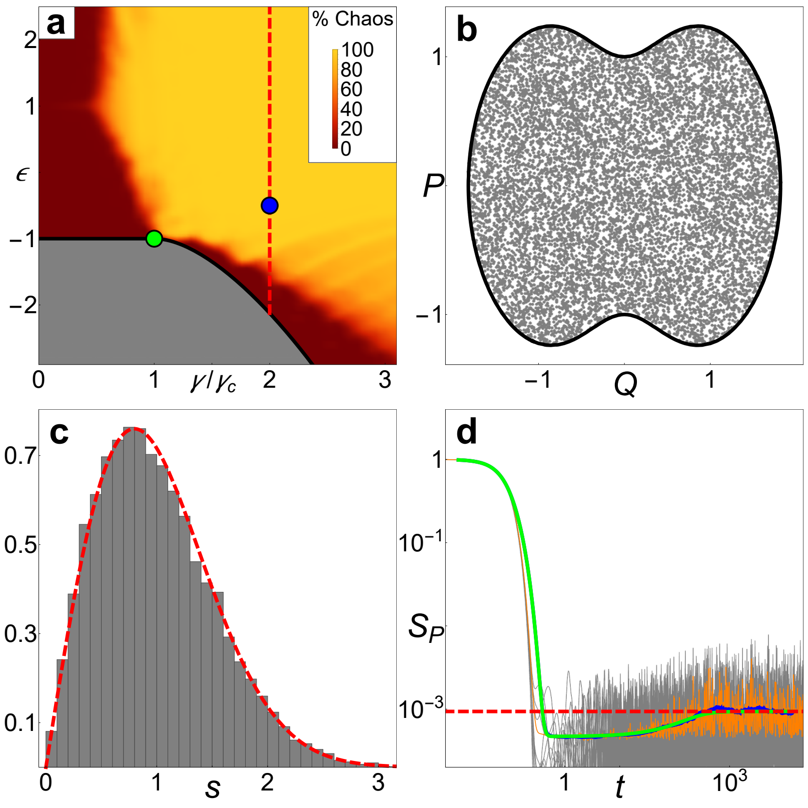

Supplementary Figure 1: Indicators of classical and quantum chaos. **(a)** Percentage of chaos over classical energy shells. The black solid line follows the ground-state energy and the vertical red dashed line marks the coupling $\gamma = 2\gamma_c$ chosen for our studies. The green dot marks the separation between the normal and the superradiant phase for the ground state. The blue dot represents the energy $\epsilon = -0.5$ used in the indicators (b) and (d). **(b)** Poincaré section ($p = 0$) for the rescaled classical Hamiltonian h_{cl} at energy $\epsilon = -0.5$. **(c)** Level spacing distribution of the unfolded spectrum (shaded area) for 22458 levels in the energy region $\epsilon \in (-1, 1.755)$ and Wigner surmise (red dashed line), $j = 100$. **(d)** Survival probability for an ensemble of 500 random states (gray solid lines) centered at energy $\epsilon = -0.5$, ensemble average (orange solid line), running average (blue solid line), analytical curve from the random matrix theory (green solid line), and the asymptotic value (horizontal red dashed line) ($j = 100$).

Supplementary Note 2: All eigenstates exhibit scars

Supplementary Fig. 2 shows the Husimi distributions of 160 eigenstates projected over the (Q, P) plane for $j = 100$. The eigenstates are selected from a list of 16,000 eigenstates with energies between $\epsilon_{\text{GS}} = -2.125$ and $\epsilon = 0$, sampled in steps of 100 from $k = 100$ to $k = 16000$. The values of the localization measure $\mathfrak{L}_k$ are indicated in the panels. We select $\gamma = 2\gamma_c$, so all these eigenstates are located in the red dashed line of Supplementary Fig. 1 (a). States with $k \leq 800$ ($\epsilon_k \leq -1.6$) are in the regular region, those with $800 < k \leq 5600$ ($-1.6 < \epsilon_k \leq -0.82$) in the mixed region, and those with $k > 5600$ ($\epsilon_k > -0.82$) are in the region of strong chaos. In all projections, the Husimi distributions display ellipsoidal shapes that can be associated with periodic orbits in the classical limit once they are identified.

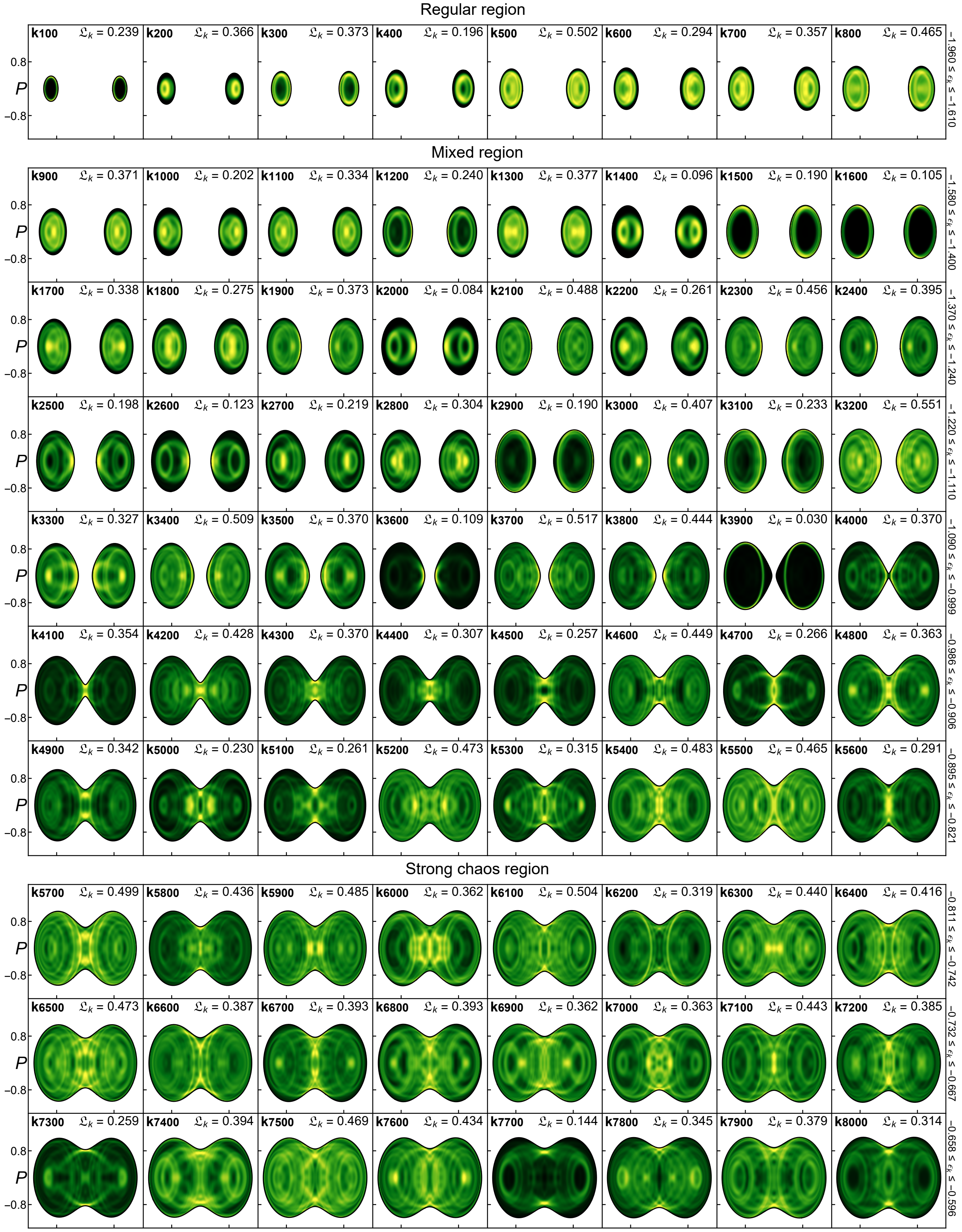

Figure continues on the next page

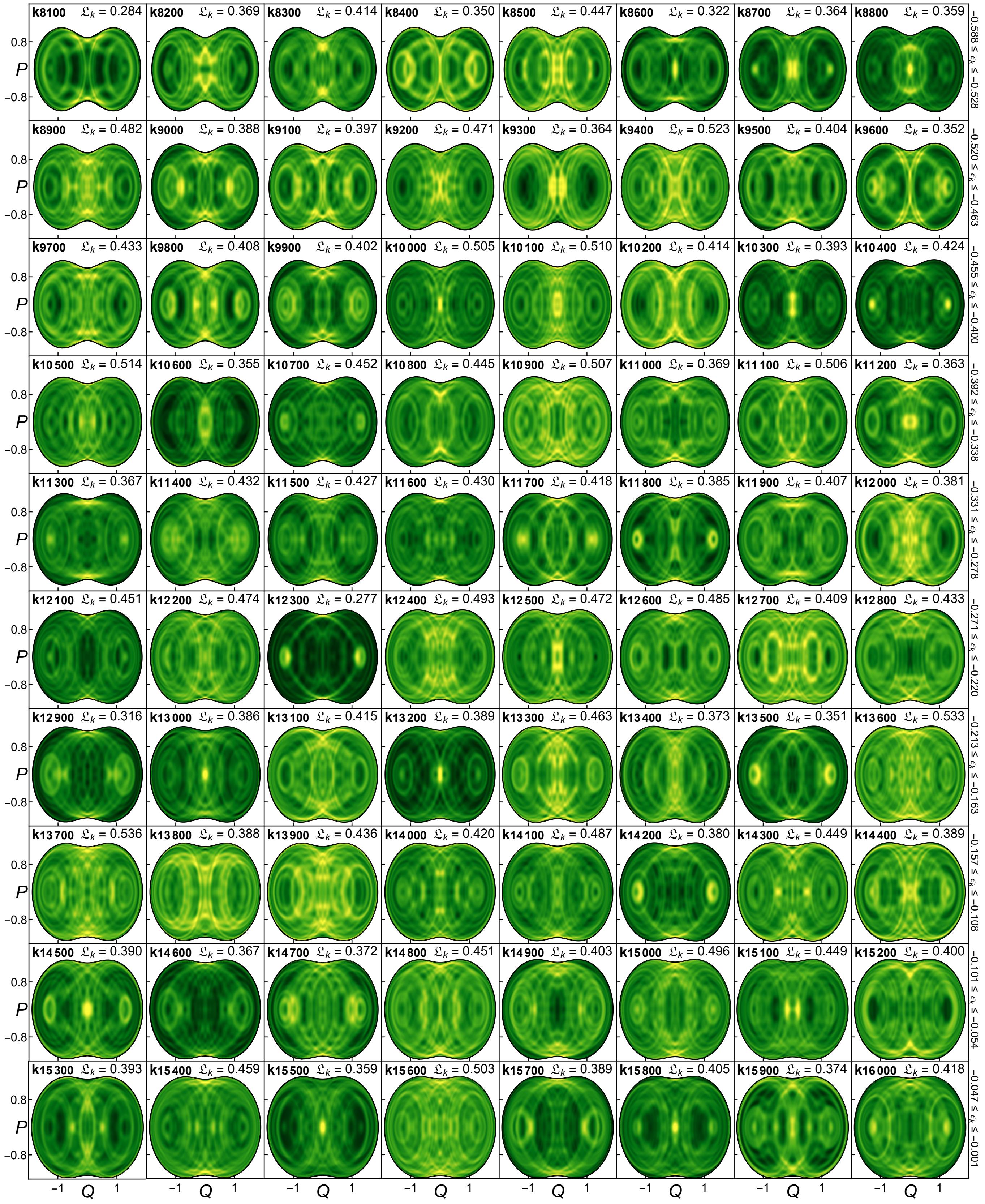

Supplementary Figure 2: Scars in all eigenstates. Husimi projections $\widetilde{\mathcal{Q}}_k$ of 160 eigenstates for $j = 100$. The values of k are indicated in the top left of each panel, along with the value of $\mathfrak{L}_k$. The energy range is indicated on the right side of each row of panels. Lighter colors indicate higher concentrations, while black corresponds to zero.

Supplementary Note 3:
Dependence on system size

The Husimi distributions for some representative eigenstates are shown in Supplementary Fig. 3 for $j = 30$ and $j = 100$. The patterns marking the periodic orbits are very similar. For each column, compare the top state ($j = 30$) with the bottom state ($j = 100$). They have very similar patterns, but the lines become better defined as j increases. As the system size increases, more lines also appear, because more periodic orbits scar the states.

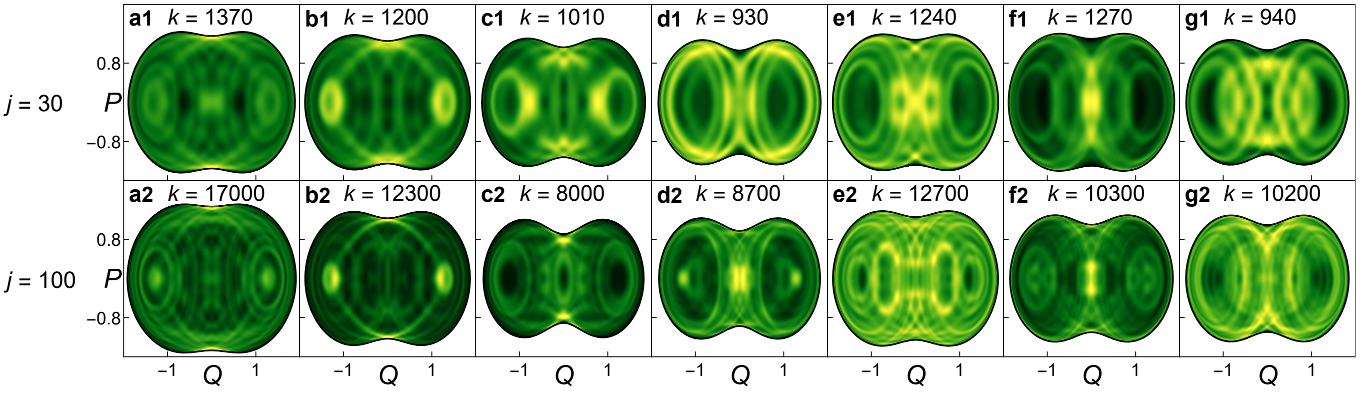

Supplementary Figure 3: Husimi projections vs. system size. Husimi projections $\widetilde{\mathcal{Q}}_k$ of 7 eigenstates for $j = 30$ (first row) and $j = 100$ (second row). Lighter colors indicate higher concentrations, while black corresponds to zero. The lines marking the periodic orbits become better defined as the system size increases.

[1] C.H Lewenkopf, M.C Nemes, V Marvulle, M.P Pato, and W.F Wreszinski, "Level statistics transitions in the spin-boson model," Phys. Lett. A **155**, 113 – 116 (1991).

[2] Clive Emary and Tobias Brandes, "Quantum chaos triggered by precursors of a quantum phase transition: The Dicke model," Phys. Rev. Lett. **90**, 044101 (2003).

[3] Clive Emary and Tobias Brandes, "Chaos and the quantum phase transition in the Dicke model," Phys. Rev. E **67**, 066203 (2003).

[4] M. A. Bastarrachea-Magnani, S. Lerma-Hernández, and J. G. Hirsch, "Comparative quantum and semiclassical analysis of atom-field systems. ii. Chaos and regularity," Phys. Rev. A **89**, 032102 (2014).

[5] Miguel Angel Bastarrachea-Magnani, Baldemar López del Carpio, Sergio Lerma-Hernández, and Jorge G Hirsch, "Chaos in the Dicke model: quantum and semiclassical analysis," Phys. Scripta **90**, 068015 (2015).

[6] M. A. Bastarrachea-Magnani, B. López-del-Carpio, J. Chávez-Carlos, S. Lerma-Hernández, and J. G. Hirsch, "Delocalization and quantum chaos in atom-field systems," Phys. Rev. E **93**, 022215 (2016).

[7] J. Chávez-Carlos, M. A. Bastarrachea-Magnani, S. Lerma-Hernández, and J. G. Hirsch, "Classical chaos in atom-field systems," Phys. Rev. E **94**, 022209 (2016).

[8] Jorge Chávez-Carlos, B. López-del Carpio, Miguel A. Bastarrachea-Magnani, Pavel Stránský, Sergio Lerma-Hernández, Lea F. Santos, and Jorge G. Hirsch, "Quantum and classical Lyapunov exponents in atom-field interaction systems," Phys. Rev. Lett. **122**, 024101 (2019).

[9] T. Guhr, A. Müller-Groeling, and H. A. Weidenmüller, "Random matrix theories in quantum physics: Common concepts," Phys. Rep. **299**, 189 (1998).

[10] S. Lerma-Hernández, D. Villaseñor, M. A. Bastarrachea-Magnani, E. J. Torres-Herrera, L. F. Santos, and J. G. Hirsch, "Dynamical signatures of quantum chaos and relaxation time scales in a spin-boson system," Phys. Rev. E **100**, 012218 (2019).

[11] David Villaseñor, Saúl Pilatowsky-Cameo, Miguel A Bastarrachea-Magnani, Sergio Lerma, Lea F Santos, and Jorge G Hirsch, "Quantum vs classical dynamics in a spin-boson system: manifestations of spectral correlations and scarring," New J. Phys. **22**, 063036 (2020).

[12] Luc Leviandier, Maurice Lombardi, Rémi Jost, and Jean Paul Pique, "Fourier transform: A tool to measure statistical level properties in very complex spectra," Phys. Rev. Lett. **56**, 2449–2452 (1986).

[13] Joshua Wilkie and Paul Brumer, "Time-dependent manifestations of quantum chaos," Phys. Rev. Lett. **67**, 1185–1188 (1991).

[14] Y. Alhassid and R. D. Levine, "Spectral autocorrelation function in the statistical theory of energy levels," Phys. Rev. A **46**, 4650–4653 (1992).

[15] E. J. Torres-Herrera and L. F. Santos, "Dynamical manifestations of quantum chaos: Correlation hole and bulge," Phil. Trans. R. Soc. A **375**, 20160434 (2017).
